# Hypoadiponectinemia in Extremely Low Gestational Age Newborns with Severe Hyperglycemia – A Matched-Paired Analysis

**DOI:** 10.1371/journal.pone.0038481

**Published:** 2012-06-05

**Authors:** Andre Oberthuer, Fatma Dönmez, Frank Oberhäuser, Moritz Hahn, Marc Hoppenz, Thomas Hoehn, Bernhard Roth, Matthias Laudes

**Affiliations:** 1 Neonatal and Pediatric Intensive Care Unit, University of Cologne, Children’s Hospital, Cologne, Germany; 2 Neonatal and Pediatric Intensive Care Unit, Children's Hospital of the City of Cologne, Cologne, Germany; 3 Department of Internal Medicine I, University of Cologne, Cologne, Germany; 4 Institute of Medical Statistics, Informatics and Epidemiology, University of Cologne, Cologne, Germany; 5 Neonatology and Pediatric Intensive Care Medicine, Department of General Pediatrics, Heinrich-Heine-University, Duesseldorf, Germany; 6 Department of General Internal Medicine, University Hospital Schleswig-Holstein, Kiel, Germany; University of Colorado Denver, United States of America

## Abstract

**Background:**

Hyperglycemia is commonly observed in extremely low gestational age newborns (ELGANs) and is associated with both increased morbidity and mortality. The objective of this study was to examine the relationship between neonatal hyperglycemia and adiponectin levels in ELGANs.

**Methodology/Principal Findings:**

Ten preterm infants between 22+6/7 and 27+3/7 weeks’ gestation with neonatal hyperglycemia (defined as pre-feeding blood glucose levels above 200mg/dl on two consecutive measurements with a maximum parenteral glucose infusion of 4mg/kg*min^−1^) formed the case cohort of this study. To every single patient of this case cohort a patient with normal fasting ( = pre-feeding) blood glucose levels was matched in terms of gestational age and gender. Adiponectin ELISAs were performed both at onset of hyperglycemia and at term-equivalent age.

In the case cohort 9/10 patients had to be treated with insulin for 1–26 days (range 0.01–0.4 IU/kg*h^−1^). Compared to matched-paired controls, significant hypoadiponectinemia was observed at onset of hyperglycemia in these affected patients (6.9µg/ml versus 15.1µg/ml, p = 0.009). At term equivalent age, normoglycemia without any insulin treatment was found in both groups. Moreover, adiponectin levels at that time were no longer significantly different (12.3µg/ml versus 20.0µg/ml; p = 0.051) possibly indicating a mechanistic relevance of this adipokine in regulating insulin sensitivity in ELGANs.

**Conclusions/Significance:**

Decreased circulating adiponectin levels are correlated with hyperglycemia in ELGANs and may contribute to the pathogenesis of impaired glucose homeostasis in these infants. These findings suggest that adiponectin might be a potential future drug target for the potentially save treatment of hyperglycemia in pre-term infants.

## Introduction

In extremely low birth weight (ELBW) premature infants and extremely low gestational age newborns (ELGANs), respectively, hyperglycemia is a commonly observed symptom and is associated with both increased morbidity and mortality [1], [2]. While elevated blood glucose levels in these infants may be triggered by high parenteral glucose administration, treatment with corticosteroids and episodes of septic infection, the underlying mechanisms evoking hyperglycemia in ELGANs often remain unclear. Currently, besides caloric restriction, the only established therapeutic approach is administration of insulin, which may entail potentially dangerous hypoglycemic episodes [3]. Identifying factors that trigger hyperglycemia in premature born babies might therefore form the basis of improved future therapeutic approaches to these babies.

In adults, the adipocytokine adiponectin enhances insulin sensitivity and plays a pivotal role in the development of type 2 diabetes [4]. Intriguingly, circulating adiponectin levels in preterm infants are substantially decreased as compared to term infants [5] and analyses at term-equivalent age have shown a sustained decrease of serum adiponectin concentrations of preterm infants in comparison to term-born babies [6]. Since both premature birth and small size at birth is a risk factor for developing type 2 diabetes in later life [7], [8], adiponectin might be a factor potentially linking prematurity and adult metabolic disease.

To approach the question if circulating adiponectin levels may influence neonatal glucose homeostasis in preterm infants, we performed a matched-paired analysis in 20 ELGANs and compared adiponectin levels in infants who had hyperglycemia in the first weeks of life with preterm infants with unimpaired glucose homeostasis who were matched in terms of gestational age and gender. Intriguingly, we found that ELGANs who experienced hyperglycemia in the first weeks of life had significantly decreased adiponectin serum levels as compared to normoglycemic matched-paired infants.

## Methods

### Objectives

The objective of this study was to examine the relationship between neonatal hyperglycemia and adiponectin levels in ELGANs. Thereby, we sought to test the hypothesis that ELGANs with impaired glucose homeostasis had significantly decreased circulating adiponectin levels as compared to normoglycemic matched-paired ELGANs.

### Participants

The study comprised 20 infants born before 28 full weeks of gestation (range 22 6/7 to 27 3/7 weeks’ gestation) enrolled at two institutions in Cologne. Gestational age was estimated from the last menstrual period and this was supported by fetal ultrasound measurements. Patients were eligible for inclusion into the case cohort of the study if fasting ( =  pre-feeding) blood glucose levels were above 200mg/dl on two consecutive measurements with a maximum parenteral glucose infusion of 4mg/kg*min^−1^ and without any infusion of lipids. Patients were eligible for inclusion into the control cohort if they had fasting blood glucose levels <180mg/dl without any insulin medication and could form a matched pair with a patient of the case cohort in terms of gestational age (±1 week) and gender. Exclusion criteria for both cohorts were clinical signs of infection or elevated C-reactive protein (>3mg/dl) or blood Interleukin-6 (>50ng/l) values or systemic steroid therapy.

### Ethics

The study was approved by the ethics’ committee of the University of Cologne, Germany. Written informed consent was obtained from parents or legal guardians of every participant. All patients participated on a completely voluntary basis.

### Description of Procedures or Investigations undertaken

In the case cohort, blood samples for adiponectin measurement were obtained within 24 hours after onset of hyperglycemia. In the control cohort, blood samples were obtained at an age corresponding to the chronological age (±1 week) on which adiponectin measurements were performed in the corresponding matched case patient. In both cohorts, a second adiponectin sample was obtained at term-equivalent age (±1 week) and serum adiponectin concentrations were determined as indicated below. For all patients ≥23 0/7 weeks’ gestation, birth weight was jugded to be appropriate for gestational age (AGA) or small for gestational age (SGA) according to the percentile values of the body weight of newborn infants by Voigt and colleagues [9]. In the case cohort, one patient was SGA with a birth weight matching the 9^th^ percentile, while all other patients had birth weights > the 10^th^ percentile.

### Enzyme-Linked Immunosorbent Assays (ELISA)

Patients’ sera were separated by centrifugation immediately after the blood samples were obtained and were kept at –20°C until analysis. Total serum adiponectin levels were determined by ELISA using the Human Adiponectin ELISA Kit from BioVendor (Czech Republic) as duplicate measurements with 10µl of patient’s serum according to the manufacturer’s protocol (analytical sensitivity: 0.47 ng/ml; intra-assay variability: 3.9%; inter-assay variability: 6.0%). Leptin levels were determined as duplicate measurements using kits from IBL international (Germany; analytical sensitivity: 1.0 ng/ml) according to the manufacturer’s protocol.

### Statistical Methods

Statistical analysis was done using IBM SPSS Statistics Version 19. Circulating adiponectin levels of matched pairs were compared by Wilcoxon’s signed-rank test both as measured and after adjustment by dividing patient’s adiponectin values to their individual body weight.

## Results

This study comprised a cohort of 20 ELGANs, of whom ten presented with severe fasting ( =  pre-feeding) hyperglycemia (>200mg/dl) in the first weeks of life (case cohort). Every single patient of the case cohort was matched by the criteria gestational age and gender to a single patient with unimpaired glucose homeostasis (control cohort). A summarized comparison of both cohorts is shown in table 1. In both cohorts an equal distribution of male and female patients (n = 5 each) and a comparable distribution of co-morbidities was observed. Detailed clinical covariates of all patients are summarized in Table S1.

**Table 1:** Patients’ demographic criteria.

		date of first measurement		term-equivalent age	
		Control Cohort	Case Cohort	Control Cohort	Case Cohort
**patients**	numbers	10	10		
	twins	5	5		
	Male	5	5		
**gestational age [weeks]**	Mean	24	24,1		
	Standard Deviation	1,2	1,8		
	Minimum	22	22		
	Maximum	26	27		
**age at measurement [d]**	Mean	17,4	17,1	102	105
	Minimum	6	6	76	68
	Maximum	47	47	123	128
**weight at measurement [g]**	Mean	706,8	642,4	2318,5	2170
	Standard Deviation	165,6	110,4	237,4	430,2
	Minimum	580	438	1970	1390
	Maximum	1160	820	2730	2690
**serum adiponectin levels**	Mean	15,1	6,9	20	12,3
	Standard Deviation	9,7	4,7	10,2	4,6
	Minimum	3,6	2,6	9,9	7,5
	Median	12,3	4,9	18,6	10,8
	Maximum	27,5	15,1	35,3	20,2
**co-morbidity**	IVH °I-II	5	2		
	IVH °III-IV	2	1		
	BPD	4	3		
	NEC °III or higher	1	0		
	ROP> = °III	0	2		
	PVL	0	1		

Comparison of clinical and demographic parameters of patients and serum adiponectin levels of the control and the case cohort of the study.

**Figure 1:**
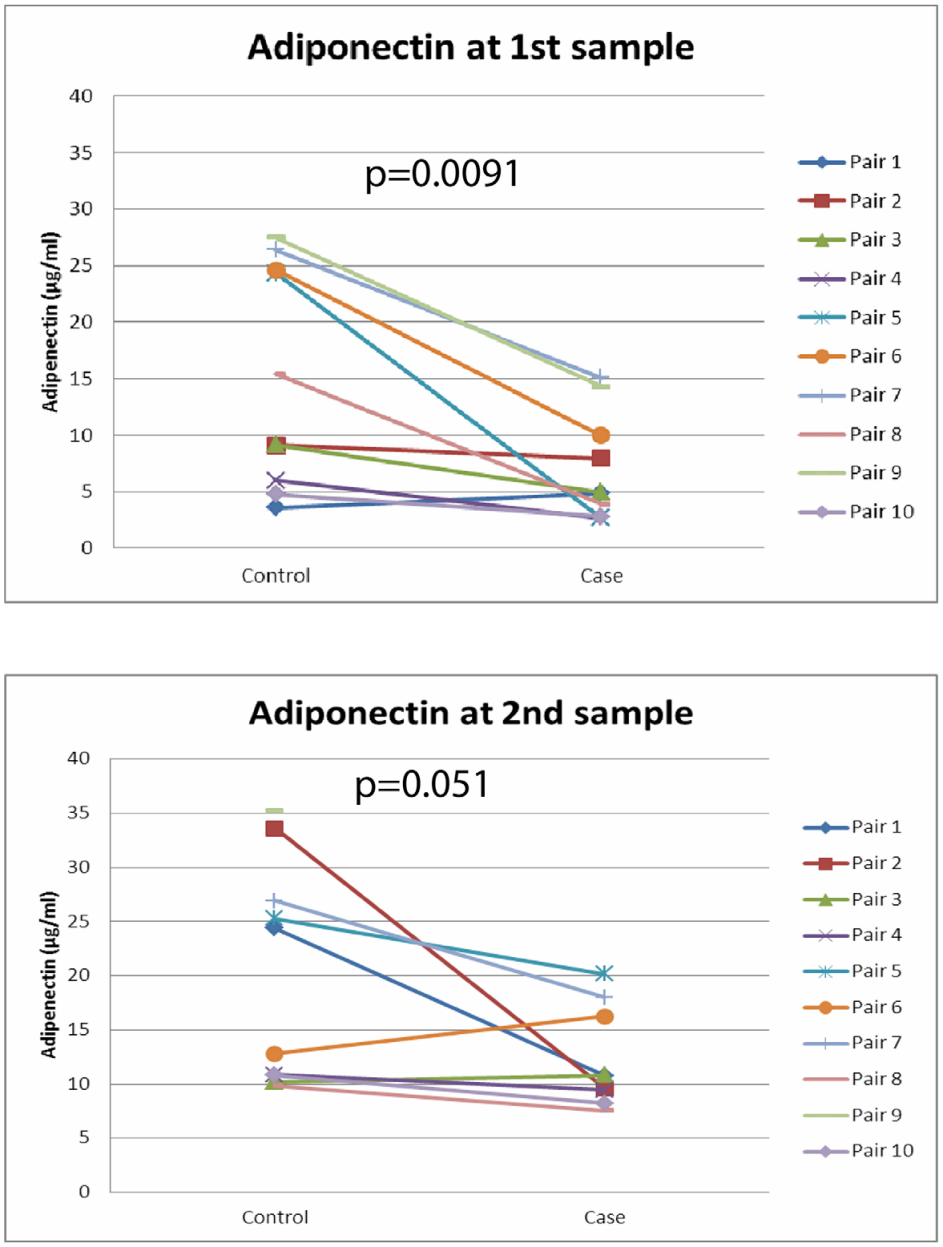
Serum adiponectin levels of ELGANs with and without hyperglycemia. Figure 1 indicates serum adiponectin levels both at the time of the first analysis (onset of hyperglycemia (A) and at term-equivalent age (B). Serum adiponectin concentrations of patients with hyperglycemia (case cohort) are shown on the right, those of patients with unimpaired glucose homeostasis are indicated on the left. Matched-pairs of patients are indicated by identical symbols and are connected by a solid line. In figure 1B, no second adiponectin value is indicated for the case patient of pair #9, as no second adiponectin sample was obtained for this patient.

In the case cohort, nine of ten patients had to be treated with insulin for 1–26 days (range 0.01–0.4 IU/kg*h^−1^; Table S1). Mean circulating adiponectin concentrations at the time of the first measurement (onset of hyperglycemia) in this cohort were 6.9 µg/ml (range 2.6 µg/ml –15.1 µg/ml). In comparison, patients of the control cohort had mean adiponectin levels of 15.1 µg/ml (range 3.6 µg/ml –27.5 µg/ml, Table 1). As shown in figure 1, the differences in serum adiponectin concentrations between both cohorts was of high statistical significance (p = 0.0091). Moreover, this difference remained statistically significant after adjusting adiponectin values to patients’ individual body weight (p = 0.013). In addition, it was noted that except for a single pair (pair #1), all patients who experienced an episode of hyperglycemia had lower serum adiponectin values than their matched control counterparts (Figure 1, Table S1), even if twins formed a matched pair. In contrast, serum leptin concentrations at the time of hyperglycemia were universally low in both the case and the control cohort patients (<0.005ng/ml; below the detectable limit of the ELISA; data not shown).

As adiponectin levels of preterm infants have been shown to rise with increasing postnatal age [5], adiponectin levels were also measured at term-equivalent age. Although no patient in either group required insulin treatment at term-equivalent age, mean adiponectin concentrations in the case cohort were still lower than in the control cohort (12.3 µg/ml (range 7.5 µg/ml –20.2 µg/ml) versus 20.0 µg/ml (range 9.9 µg/ml –35.5 µg/ml)). Although this result had a strong trend towards statistical significance (p = 0.051, Figure 1, Table 1), this observation was not significant after adjusting for patient’s body weight (p = 0.11).

## Discussion

Recent studies have shown that both babies who are small for gestational age and preterm infants have lower circulating adiponectin levels than appropriate for gestational age and term-born babies, respectively [5], [10]. While hypoadiponectinemia has been shown to be associated with type 2 diabetes in adults [4], it is unclear if low circulating adiponectin levels in preterm infants contribute to the often impaired glucose tolerance of these babies. Furthermore, it is unclear if adiponectin is linked to the increased risk of premature or SGA infants to develop type 2 diabetes in later life. In this study, we for the first time performed a matched-paired analysis of a rare cohort of extremely premature infants to compare serum adiponectin levels in ELGANs with hyperglycemia in the first weeks of life to ELGANs with unimpaired glucose homeostasis.

The central finding of our study was that very premature infants with severe hyperglycemia had significantly lower circulating adiponectin levels although leptin levels were univserally low in both cohorts. It is tempting to hypothesize this observation might indicate a mechanistic relevance of adiponectin in regulating insulin sensitivity in preterm neonates. However, we think that our data do not yet allow to conclude that adiponectin is mechanistically responsible for hyperglycemia in extreme premature infants. In contrast, it appears just as plausible that hyperglycemia and low plasma adiponectin levels represent immaturity of beta cells and adipose tissue, respectively, rather than being causally associated. Future controlled trials are therefore needed to assess the impact of adiponectin plasma levels on the glucose homeostasis of very premature infants. If it can be proven that adiponectin is involved in the pathogenesis of neonatal hyperglycemia, it appears reasonable to consider inducing adiponectin production, e.g. by pharmacologic interventions, as a future treatment option for hyperglycemia in without the risk of hypoglycemic events that come along with the administration of insulin [3]. However, experience with currently available substances that raise adiponectin levels, such as thiazolidinediones, appear not to warrant the use of these drugs in ELGANs. Alternatively, since adiponectin has been shown to be absorbed from breast milk in a biologically active form [11], our data might further support feeding breast-milk to ELGANs to positively affect the metabolism of these infants. In this context, it is remarkable, that a previous studies by Martin and colleagues showed a positive correlation of breast milk adiponectin concentrations with mothers’ body mass index (BMI) despite the known inverse correlation of adiponectin serum levels with BMI [12], possibly indicating additional protective effects of breast milk on the metabolic situation of newborn infants.

Similar to a recent trial by Saito and colleagues [13], who described that serum adiponectin levels of preterm infants increased from the first day of life to term-equivalent age, we also observed a rise in circulating adiponectin levels from the time of the first measurement to term-equivalent age both in the case and the control cohort (Table S1). However, we also noted a few patients in the control cohort with lower or equal serum adiponectin levels at term-equivalent age (n = 2 and n = 1, respectively). In contrast to the study by Saito, adiponectin levels in these patients were not determined on the first day of life but at 24 and 25 days of life, respectively. Possibly, adiponectin levels in these patients had already increased from birth to the time of the first measurement and had reached a steady state on a sufficiently high level, in particular since no hyperglycemia was noted in these patients. In this context, it is also important to note that we did not observe an absolute adiponectin cutoff-level below which hyperglycemia universally occured. This phenomenon is most likely attributed to the age-dependent rise in circulating adiponectin concentrations, due to which adiponectin cut-off values may differ depending on patients’ age. This hypothesis is supported by the observation that the lowest adiponectin values in the control cohort were observed in those patients who were the youngest at the time of the analysis.

### Limitations

An important limitation to our study is the low absolute number of patients. Although we observed that circulating adiponectin levels in ELGANs with hyperglycemia were significantly lower than in infants who were normoglycemic, the low number of patients in our study impedes a clear interpretation of the differences in adiponectin levels between the two cohorts at term-equivalent age. While it is intriguing that adiponectin levels of patients with hyperglycemia tended to remain lower at term-equivalent age than in control patients, this finding only had a trend towards statistic significance (p = 0.051). Therefore, analyses of adiponectin levels in larger cohorts of patients are needed to determine if hypoadiponectinemia persists at term-equivalent age in patients who suffered from hyperglycemia in the first weeks of life. It will be interesting to observe if this trend can be confirmed and whether low adiponectin levels in patients who experienced hyperglycemia in early life persist through future life. However it also needs to be stressed that the patient cohort in this study is extremely rare and that recruitment of these 20 patients had to be performed over a 24-month period in two large neonatal intensive care units in Germany. In view of this difficulty it also appears reasonable that the pre-feeding blood glucose cutoff value was set to <180mg/dl for the control group and not to an even stricter value, such as 150mg/dl. The finding of significantly different serum adiponectin values in both cohorts retrospectively justifies the chosen cutoff for the control cohort.

Future studies should address the question if infants with hypoadiponectinemia in the first weeks of life are at an increased risk of developing metabolic disease, such as type 2 diabetes, in later life. If so, determining neonatal adiponectin levels could serve as a risk predictor for metabolic disease in later life and could eventually legitimate early dietary or even pharmacologic treatment with either adiponectin inducing agents or substances that mimic adiponectin effects.

## Supporting Information

Table S1 **Serum adiponectin concentrations and clinical co-variates of patients who participated in the present study.** In addition to anthropometric parameter, adiponectin values, blood glucose levels, insulin requirements, the center, in which the patient was treated (1 =  University of Cologne Children’s Hospital; 2 =  Children’s Hospital of the City of Cologne) and information on both the reason for prematurity and co-morbidities are indicated (IVH  =  intraventricular hemorrhage; PVL  =  periventricular leukomalacia; BPD  =  bronchopulmonary dysplasia; ROP  =  retinopathy of prematurity; NEC  =  necrotizing enterocolitis). A value of −9999 corresponds to missing information on this parameter.
(XLS)
